# Attentional Networks in Parkinson’s Disease

**DOI:** 10.3233/BEN-129020

**Published:** 2013

**Authors:** Chiara Cristinzio, Monica Bononi, Sylvie Piacentini, Alberto Albanese, Paolo Bartolomeo

**Affiliations:** ^1^Dipartimento di Psicologia, Università Cattolica del Sacro Cuore, MilanItaly; ^2^Carlo Besta Neurological Institute, MilanItaly; ^3^Istituto di Neurologia, Università Cattolica del Sacro Cuore, RomeItaly; ^4^dINSERM – UPMC UMRS 975, Brain and Spine Institute, Groupe Hospitalier Pitié-Salpêtrière, ParisFrance; ^5^AP-HP, Groupe Hospitalier Pitié-Salpêtrière, Fédération de Neurologie, ParisFrance

**Keywords:** Attention, alerting, orienting, executive control

## Abstract

We tested the efficiency of three attentional systems (spatial orienting, phasic alerting and executive control) in patients with Parkinson’s disease (PD), by using a modified version of the Attention Network Test, which employs acoustic tones to modulate phasic alertness. PD patients were generally slower than age-matched controls, but they showed a similar pattern of effects and interactions. Responses were faster with congruent than with incongruent stimuli (executive control), with valid visual cues than with invalid or no cues (orienting), and when acoustic tones preceded the target (alerting). This last effect was significantly larger in PD patients than in controls. We concluded that, for the present group of patients, the activity of attentional networks was relatively normal, if slowed. Slowed responses in PD may be improved by the use of acoustic stimuli, with potential clinical implications.

## 1. Introduction

Attention is a collective term used to indicate a family of functions which allow us to pursue our goals in spite of external distractors, while remaining able to respond to unexpected, potentially dangerous events. Exploration of attentional abilities is of paramount practical importance in normal individuals and in neurological patients, for example in order to assess patients' capacity for independent life, to determine driving license retainment or cancellation, or to prescribe rehabilitation procedures.

In the early stages of Parkinson's Disease (PD), attentional deficits such as bradyphrenia [1], defined as impaired concentration and slowed cognitive processing, may not be clinically apparent, but can be detected by using specific neuropsychological tests or response time (RT) tasks. For example, even in early PD cognitive impairment can occur as deficits of attention and executive functions [2], as evaluated with standardized neuropsychological test (Digit span backward and Trail Making Test). Other findings in PD patients indicate an early impairment of attention [3]. Attentional deficits also interact with motor impairment in PD. For example, slowed RTs in computerized tasks correlate with increased frequency of falls [4]. Thus, techniques of rehabilitation focused on attention training might improve walking and prevent falls in PD patients.

Studies exploring attentional abilities in PD have principally investigated "covert" orienting of attention in space, i.e. the orienting of attention to stimuli in the absence of eye movements [5,6]. In some studies, PD patients did not show any substantial deficit of visual covert orienting [7–9], whereas others report-

ISSN 0953-4180/13/$27.50 © 2013 – IOS Press and the authors. All rights reserved ed the presence of impaired orienting of spatial attention [10–12], which can correlate with clinical severity [13]. Other studies reported a reduced or absent inhibition of return [14], a component of exogenous, or automatic, orienting consisting in a slowing of RTs to targets presented at the same location as a previous stimulus [15].

In addition to spatial orienting, two further attentional abilities are crucial to our interaction with the environment, namely alerting and executive control. Alerting prepares the system to produce rapid responses; executive control is active when the cognitive system has to resolve conflictual situations, make a decision, plan and control responses. The study of attentional control seems particularly appropriate on clinical grounds, because impaired conflict resolution has been associated with episodes of freezing of gait in PD [16], and poor control of divided attention can increase patients' difficulty in walking [17]. The Attention Network Test (ANT) [18] has been specifically devised to measure these three aspects of attention in an independent manner but within a single RT task, easy to administer in clinical settings. Subjects have to press one of two keys in response to the orientation of an arrow flanked by distractor arrows, which can point either to the same direction as the target arrow (congruent condition), or to the opposite direction (incongruent condition). The congruent – incongruent RT difference provides a measure of executive control in conflict resolution. The arrow can be presented above or below fixation; before target presentation, a spatial cue can occur either at the same location as the impending target (valid condition), or at the opposite location (invalid condition); in other trials, no cue is presented. The valid – invalid RT difference provides a measure of spatial orienting, whereas the cue – no-cue RT difference estimates the effect of phasic alerting. Thus, in the original version of the ANT the same visual cues are used to test both orienting and alerting. The ANT was subsequently modified [19], in order to measure not only the independency of the three networks, but also their possible interactions. To this end, a short high-frequency tone is added to half of the trials, prior to cue and target presentation. The difference between tone and no-tone RTs provides a measure of phasic alerting; this measure is now independent of the spatial orienting assessed by the visual cue. We took advantage of the modified version of the ANT, which has proved itself useful to study attentional deficits in neurological patients [20], to explore attentional abilities in PD patients.

## 2. Methods

### 2.1. Participants

Fourteen PD patients connected to the outpatient department of the Carlo Besta Neurological Institute in Milan, Italy (nine men and five women), gave informed consent to participate in the experiment. All patients received pharmacological therapy and were under medication during the testing sessions. Before experimental testing, all patients underwent the Unified Parkinson's Disease Rating Scale (UPDRS) motor evaluation and a standard neuropsychological evaluation. Thirteen healthy participants (seven men and six women) recruited among patients' relatives also performed the test. Control subjects had no neurological or psychiatric disorders and were matched to the patients for age (patients: mean age 62.7 years, range 51–78; controls: mean age 57.1 years, range 45–70; $t = 1.74$; $p = 0.09$) and educational level (patients: 11.7 years of schooling, range 8–17; controls: 12.3 years, range 8–17; $t = -1.05$; $p = 0.29$). All subjects gave informed written consent to participate in the experiment. Table 1 reports patients' characteristics of and the results of motor and cognitive evaluation.

### 2.2. Procedure

Each trial began with a fixation cross (Fig. 1), which remained on the screen for a duration variable from 400 to 1600 ms. On half of trials, a brief (50 ms) acoustic alerting signal consisting of a 2000-Hz tone was presented after the fixation period. After 400 ms, an orienting cue, consisting of an asterisk, was presented for 50 ms above or below the fixation point on 2/3 of the trials. After another 50 ms, the target and flankers were presented either in the same location as the previous orienting cue, or in the opposite location. The target consisted of a horizontal arrow that could point either to the left or to the right. The target arrow was flanked by four arrows pointing either in the same direction as the target or in the opposite direction. The length of each arrow was 0.55°; they were separated by 0.06° spaces. The target remained on the screen until response or 3000 ms had elapsed. The target screen was followed by the fixation cross, which was displayed for a variable duration dependent on the duration of the initial fixation point and on the participant's RT, so that each trial had a constant duration of 4600 ms.

Participants were seated approximately 53 cm from the computer screen, and were instructed to respond as

Table 1
Characteristics of patients group and results of cognitive tests

| Patient | Age | Education (years) | Disease duration (years) | Motor UPDRS | MODA[1] | Verbal digit span | TMT[2] | Stroop[3] | Selective attention test[4] |
|---|---|---|---|---|---|---|---|---|---|
| P01 | 54 | 13 | 6 | 12 | 92.7 | 8 (4) | 6 (4) | 12.5 (4) | 58 (4) |
| P02 | 55 | 13 | 3 | 20 | 93.6 | 7 (4) | 26 (4) | 30.5 (2) | 51 (3) |
| P03 | 77 | 5 | 2 | 20 | 96.8 | 5 (4) | 93 (2) | 25.0 (4) | 42 (3) |
| P04 | 65 | 13 | 11 | 12 | 97.3 | 8 (4) | 4 (4) | 26.5 (4) | 59 (4) |
| P05 | 69 | 13 | 2 | 21 | 97.5 | 6 (4) | 69 (3) | 25.0 (4) | 45 (2) |
| P06 | 69 | 8 | 30 | 16 | 90.0 | 4 (2) | 141 (1) | 49.0 (0) | 44 (2) |
| P07 | 78 | 8 | 4 | 29 | 93.0 | 5 (4) | 69 (3) | 27.5 (4) | 54 (4) |
| P08 | 65 | 8 | 12 | 11 | 94.6 | 5 (4) | 69 (3) | 25.0 (4) | 49 (4) |
| P09 | 52 | 8 | 3 | 26 | 99.9 | 5 (3) | 57 (4) | 18.0 (4) | 52 (3) |
| P10 | 64 | 17 | 10 | 10 | 94.9 | 7 (4) | 49 (4) | 13.5 (4) | 55 (3) |
| P11 | 62 | 13 | 2 | 15 | 96.9 | 6 (4) | 39 (4) | 16.0 (4) | 56 (4) |
| P12 | 62 | 13 | 3 | 17 | 96.5 | 6 (4) | 50 (4) | 18.0 (4) | 57 (4) |
| P13 | 55 | 13 | 10 | 15 | 95.8 | 7 (4) | 74 (3) | 20.5 (4) | 59 (4) |
| P14 | 51 | 10 | 13 | 27 | 96.9 | 6 (4) | 55 (4) | 10.5 (4) | 59 (4) |

Normative scores, ranging from 4 (normal) to 0 (pathological), are reported in parentheses. 1 Milan Overall Dementia Assessment (24); scores $> 89.0$ are considered normal. 2 Trail Making Test; scores for part B adjusted for part A. 3 Score for the third part corrected for the score for the second part. 4 Attentional test consisting in cancelling target digits embedded among other numbers that serve as distractors.

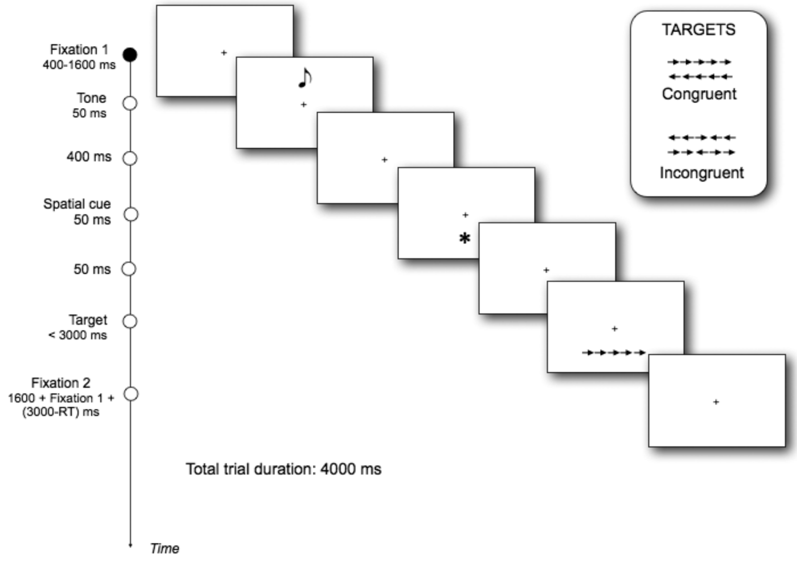

Fig. 1. Experimental procedure: example of a down valid warned congruent trial (the musical notation represents the alerting tone and was not presented visually).

rapidly and as accurately as possible to the direction of the target stimulus by pressing one of two possible keys in the keyboard. Participants were instructed to respond only to the targets and not to the orienting cues or to the alerting tones. Trials were organized in six blocks of 48 trials each and participants were allowed to rest between blocks. The six experimental blocks were preceded by 24 practice trials, in which participants received feedback concerning their speed and accuracy. Practice trials were discarded from the analysis. Each block had 4 trials per condition resulting in a total of 24 identical trials per condition. Each block lasted about 6 minutes for controls, and approximately 10 minutes for patients.

## 3. Results

### 3.1. Response times

RT outliers were discarded from analysis by excluding RTs faster than 100 ms or slower than 1700 ms. This resulted in the exclusion of 0.7% of the trials for controls and 1.3% for PD patients. Table 2 reports the

Table 2

Patients' and controls' mean response times with SDs (in ms) and proportion of correct responses (in parentheses) for each experimental condition

| | Congruent distractors | | | | | | Incongruent distractors | | | | | |
|---|---|---|---|---|---|---|---|---|---|---|---|---|
| | No Tone | | | Tone | | | No Tone | | | Tone | | |
| Visual cue | Invalid | Absent | Valid | Invalid | Absent | Valid | Invalid | Absent | Valid | Invalid | Absent | Valid |
| Patients | $726 \pm 162$ (0.95) | $723 \pm 176$ (0.96) | $690 \pm 153$ (0.94) | $665 \pm 160$ (0.95) | $641 \pm 158$ (0.95) | $624 \pm 130$ (0.95) | $807 \pm 150$ (0.93) | $835 \pm 179$ (0.93) | $793 \pm 177$ (0.94) | $795 \pm 160$ (0.92) | $777 \pm 161$ (0.91) | $745 \pm 143$ (0.92) |
| Controls | $564 \pm 58$ (0.99) | $576 \pm 76$ (0.98) | $534 \pm 67$ (0.98) | $551 \pm 69$ (0.99) | $533 \pm 68$ (0.99) | $513 \pm 75$ (0.99) | $648 \pm 99$ (0.94) | $676 \pm 128$ (0.94) | $615 \pm 91$ (0.97) | $653 \pm 76$ (0.96) | $637 \pm 78$ (0.97) | $612 \pm 77$ (0.97) |

correct RTs. We conducted a repeated-measure analysis of variance on mean correct RTs with three within-subject factors: congruency (2 levels: congruent, incongruent), visual cue (3 levels: valid, invalid and no cue) and acoustic tone (2 levels: tone, no tone) plus one between-subject factor, participant group (2 levels: patients and controls). As expected, patients produced slower responses than controls (main effect of group: $F(1, 25) = 9.28$; $p < 0.05$). All within-group factors, namely congruency, visual cue and acoustic cue, were significant. Subjects were faster in the condition where the target and flankers arrows pointed to the same direction (main effect of congruency, $F(1, 25) = 165.3$; $p < 0.001$). The main effect of visual cue ($F(2, 50) = 36.5$; $p < 0.001$) resulted from participant being faster to detect the target when it was presented at the same location as the cue, rather than when no cue was presented ($F(1, 25) = 52.9$; $p < 0.001$), or when the cue was presented at the opposite location ($F(1,25) = 74.2$; $p < 0.001$). The presence of the acoustic tone speeded performance ($F(1, 25) = 37.37$; $p < 0.001$), indicating an effective activation of the alerting network. There was also an interaction between tones and visual cues ($F(2, 50) = 5.6$, $p < 0.05$). When the tone was presented in association with a valid visual cue, responses were faster than in all others conditions ($F(1, 25) = 69{,}8$; $p < 0.001$). This interaction likely depended on the facilitating effect of acoustic cues on the perception of visual target (21). Importantly, there was also an interaction between group and tone ($F(1, 25) = 8.5$; $p < 0.05$), because the tone speeded up controls' performance by 19 ms, but it accelerated PD patients' performance by 54 ms (a threefold increase). No other interactions reached significance.

### *3.2. Accuracy*

Accuracy was in general good, larger than 90% (see Table 2). Proportions of correct responses were arcsin-transformed and submitted to an analysis of variance with the same factors used for the RT analysis. There was a main effect of congruency ($F\,(1, 25) = 14.5$; $p < 0.001$) and an interaction between group, congruency and acoustic cue ($F\,(1,25) = 4.6$; $p < 0.05$). Analysis of this interaction showed that when the acoustic tone was presented, PD patients had no significant difference between congruent versus incongruent stimuli ($F\,(1, 25) = 2.1$; $p = 0.1$), whereas this differences remained reliable in controls ($F\,(1, 25) = 7.8$; $p < 0.05$). No other effects or interactions reached significance. Thus, the analysis of performance accuracy seems consistent with RT results in suggesting an increased positive effect of acoustic tones in PD patients as compared to controls, who still suffered from the interference effect of incongruent flankers despite the acoustic tone.

## 4. Discussion

We tested the efficiency of spatial orienting, phasic alerting and executive control in a group of PD patients by using a modified version [19] of the Attention Network Test [18]. Previous studies using the original version of the ANT (without acoustic tones) had demonstrated deficits of spatial orienting [12] or executive control [16] in PD patients. The use of the modified version of the ANT allowed us to highlight an increased response of our PD patients to the alerting tone, in the context of the expected general slowing of patients' RTs. On the other hand, PD patients had relatively normal spatial orienting and executive control, as indicated by validity and congruency effects similar to controls'. This result may parallel the relative preservation of cognitive abilities in these patients (see Table 1); it may also depend at least in part on the fact that patients were under medication at the time of testing.

Apart form general RT slowing, the only other parameter which reliably distinguished PD patients' RT performance from controls' was patients' increased susceptibility to alerting tones. Tones were able to speed up PD patients' performance three times more than controls'. There was of course more room for improvement in patients' generally slowed RTs (likely to result at least in part from bradykinesia), than in control's performance. However, the fact that PD patients could partly overcome bradykinesia thanks to an acoustic tone is of clinical interest per se. Moreover, patients showed a normal interaction between orienting and alerting systems, thus demonstrating the ability to integrate distinct external stimulations to improve their speed of response. This result is potentially relevant for rehabilitation purposes, because it suggests that the combination of different modalities of stimulation can generate more improvement than input in a single modality. In broad agreement with the positive effect of acoustic tones observed in the present results, it has been reported that rhythmic auditory stimulation can have a significant effect on gait velocity, stride length and cadence [16]. When patients performed a training in which they walked at tempo of rhythmically accentuated music, parameters of gait significantly improved. In addition, even listening to music without walking or performing other motor exercises has been shown to improve motor function, particularly hypokinesia [22], as well as emotional functions. The effect of music can be observed in various types of abilities, such as precision of finger and arm movement tested after a music listening session [23].

In conclusion, the present results demonstrate that PD patients can have relatively normal, if slowed, efficiency of attentional networks. A limitation of the present study was the relatively small number of recruited patients, which calls for confirmation of the present findings with larger patient samples. Such follow-up studies may be clinically important to explore the potential usefulness of the positive effects of acoustic tones for rehabilitation purposes.
